# The Effects of Profile Errors of Microlens Surfaces on Laser Beam Homogenization

**DOI:** 10.3390/mi8020050

**Published:** 2017-02-13

**Authors:** Axiu Cao, Hui Pang, Jiazhou Wang, Man Zhang, Jian Chen, Lifang Shi, Qiling Deng, Song Hu

**Affiliations:** 1 Institute of Optics and Electronics, Chinese Academy of Sciences, Chengdu 610209, China; longazure@163.com (A.C.); wuli041@126.com (H.P.); ph@ioe.ac.cn (J.W.); zhangman881003@126.com (M.Z.); husong@ioe.ac.cn (S.H.); 2 University of Chinese Academy of Sciences, Beijing 100049, China; chenjian@mail.ie.ac.cn; 3 State Key Laboratory of Transducer Technology, Institute of Electronics, Chinese Academy of Sciences, Beijing 100190, China

**Keywords:** microlens array, beam homogenization, surface profile error, microfabrication

## Abstract

Microlens arrays (MLAs) are key optical components in laser beam homogenization. However, due to imperfect surface profiles resulting from microfabrication, the functionalities of MLAs in beam modulation could be compromised to some extent. In order to address this issue, the effects of surface profile mismatches between ideal and fabricated MLAs on beam homogenization were analyzed. Four types of surface profile errors of MLAs were modeled theoretically and numerical simulations were conducted to quantitatively estimate the effects of these profile errors on beam homogenization. In addition, experiments were conducted to validate the simulation results, revealing that profile errors leading to optical deviations located on the apex of microlenses affected beam homogenization less than deviations located further away from it. This study can provide references for the further applications of MLAs in beam homogenization.

## 1. Introduction

Uniform illuminations on target surfaces are required in many applications such as laser fusion, laser cosmetology and material processing [1,2,3]. However, most laser beams are distributed in the Gaussian form or other nonuniform forms. Therefore, the shaping of an arbitrary input intensity into a top-hat format is a key issue [4,5].

Diffractive and refractive solutions have been used to improve the homogeneities of the laser beams. The diffractive elements are used for beam shaping, in which certain fractions of inputs are directed with specific angles, generating outputs with desired intensity distributions. Since diffractive solutions depend heavily on the light wavelength, they are only suitable for limited applications with low rates of energy utilization [6,7]. Meanwhile, the refractive elements are suitable for a wider spectral range due to lower dispersions compared to diffractive counterparts [8,9,10]. As a key component for light refraction, a microlens array (MLA) consisting of a Fourier lens and at least one regular microlens array has been used for beam homogenization. More specifically, the input radiation is firstly divided into multiple fractions by each channel of the tandem microlens array. Then, the fractions can be superposed with each other in the focal plane of the Fourier lens to form a light with a uniform distribution of intensities [9]. 

As a key optical component requesting high-accuracy surface profiles, the fabrication of MLAs is of importance. Thermal reflow is a well-established technique of fabricating MLAs by thermally flowing pre-patterned photoresist posts [11,12]. However, this approach cannot produce MLAs with high filling factors (~78% in an orthogonal array and ~90% in a hexagonal array). When MLAs fabricated by thermal reflow are used in beam homogenization, a zero-order spot with high intensities is always generated due to limitations in filling factors.

In order to address this issue, our group developed an approach based on moving-mask exposure, enabling the fabrication of MLAs with a filling factor of 100% [13]. However, due to the nonlinear effects in photoresist exposure, there is an issue of surface profile mismatches between the ideal and fabricated MLAs, leading to non-uniform intensity distributions in beam homogenization. 

In this study, we evaluated the effects of surface profile errors on beam homogenization, including theoretical analysis, numerical simulations and experimental estimations. The manuscript is arranged as follows: Section 2 is the theoretical analysis where surface profile errors of MLAs are classified into four types. Section 3 is the numerical analysis where the beam deviations in response to four types of surface profile errors of MLAs are quantified. Section 4 is the experimental section where the results of numerical simulations are validated. Section 5 is the conclusion of this manuscript. 

## 2. Principle

**Figure 1:**
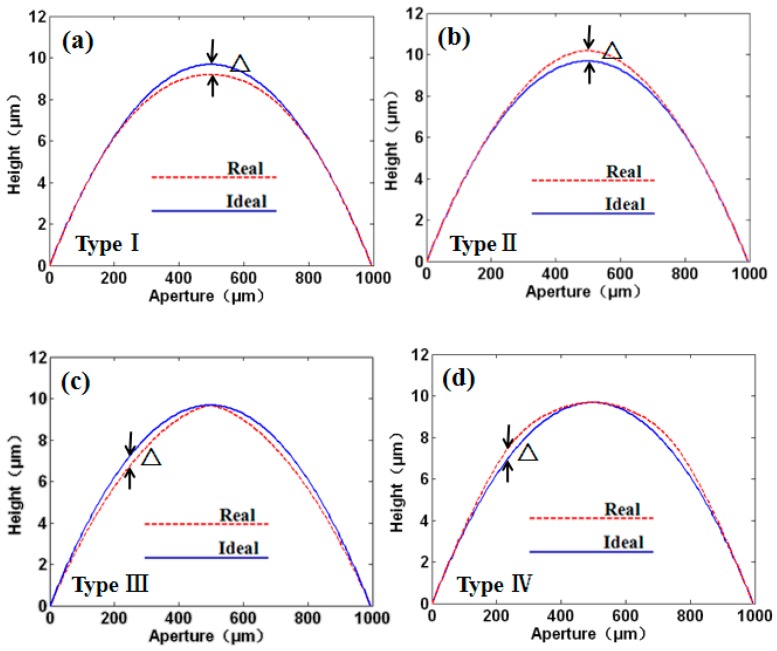
Four types of surface profile errors of microlenses: (**a**) type I; (**b**) type II; (**c**) type III; and (**d**) type IV. The dashed and solid lines represent the cross-section profiles of fabricated and ideal microlenses, respectively, where △ represents the maximal profile mismatch between the ideal and fabricated microlenses.

Most microlenses require a spherical surface where the sag height $z_{0}$ is given by Equation (1).

(1) $$z_{0}=\frac{cr^{2}}{1+\sqrt{1-c^{2}r^{2}}}$$

where *c* is the curvature (the reciprocal of the radius), and *r* is the radial coordinate in lens units. However, fabrications always lead to errors of surface profiles, which can be classified into four types (see Figure 1). The symbol △ represents the maximal profile mismatch between the designed and fabricated microlens. For type I and II errors, the maximal mismatches were located in the central part of the microlens (see Figure 1a,b), while for type III and IV errors (see Figure 1c,d), the maximal mismatches were located in about one-quarter or three-quarters of the microlens.

Based on the numerical fitting of results from multiple experiments, surface profile errors *z_i_* can be described as Equation (2).

(2) $$z_{i}=\frac{cr^{2}}{1+\sqrt{1-c^{2}r^{2}}}+\Delta_{i},i=1,2,3,4$$

where $\Delta_{i}$ (i=1,2,3,4) represents four types of surface profile errors which can be described in Equations (3)–(6), respectively.

(3) $$i=1,\Delta_{1}=-\Delta \exp(\frac{-r^{2}}{w^{2}})$$

(4) $$i=2,\Delta_{2}=\Delta \exp(\frac{-r^{2}}{w^{2}})$$

(5) $$i=3,\Delta_{3}=-\Delta \sin(\frac{r}{d/2}\pi )$$

(6) $$i=4,\Delta_{4}=\Delta \exp(\frac{-{\left(r-\frac{d}{4}\right)}^{2}}{w^{2}})$$

where *w* is the radius of the circle in which the maximal value of the surface profile error decreases to 1/e of the maximal value, and *d* is the aperture of the microlens.

## 3. Simulations

**Figure 2:**
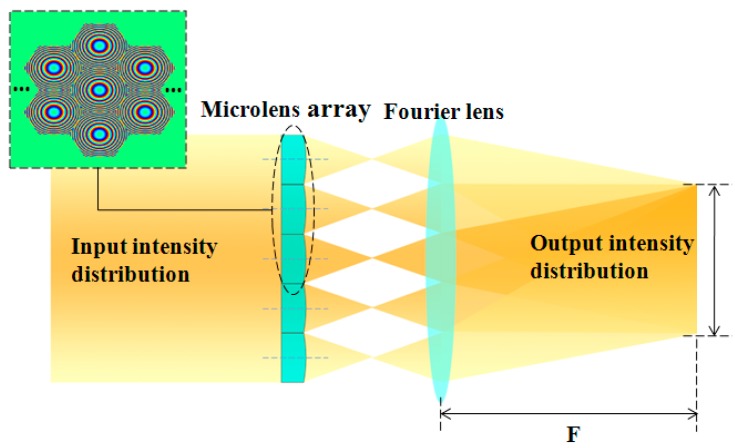
The numerical simulations of microlens array (MLA)-based beam homogenization, where an input optical beam transmits a MLA and a Fourier lens.

To analyze the effect of surface profile variations of MLAs on laser beam homogenization, numerical simulations were conducted. As shown in Figure 2, the simulations included a MLA and a Fourier lens. Based on the theory of scalar diffraction, when a laser beam transmits through a MLA and a Fourier lens in turn, the optical field at the focal plane of the Fourier lens is determined by the Fourier transformation of the transmission function of the MLA [14]. 

Firstly, the model for light field analysis and calculations based on the theory of scalar diffraction were proposed to study laser beam homogenization. The degree of beam homogenization based on the ideal spherical microlens was calculated by MATLAB (version 7.1, MathWorks, Natick, MA, USA). Then, spherical microlenses with surface profile errors were introduced to replace the ideal microlens to evaluate their effects on deviations of beam homogenization.

The key parameters used in the simulation were as follows. The light wavelength was 650 nm. The microlenses were tightly arranged in a hexagon for a filling factor of 100% (see Figure 2). The aperture of the microlens was hexagonal with a distance of 1 mm between the parallel edges. The focal lengths of the microlens and Fourier lens were 28 and 300 mm, respectively.

**Figure 3:**
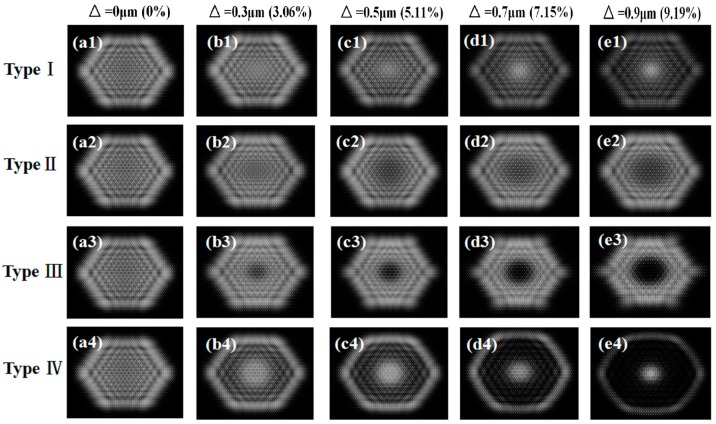
Numerical simulations of images after beam homogenization by passing MLAs with four types of surface profile errors. The maximal surface profile errors were 0 μm (0%) for (**a1**–**a4**); 0.3 μm (3.6%) for (**b1**–**b4**); 0.5 μm (5.11%) for (**c1**–**c4**); 0.7 μm (7.15%) for (**d1**–**d4**); and 0.9 μm (9.19%) for (**e1**–**e4**), respectively.

When the maximal surface profile errors were 0, 0.3, 0.5, 0.7 or 0.9 μm (the value of △ in microns has been quoted as a percentage of the microlens height), the corresponding deviation of beam homogenization was obtained, respectively (see Figure 3). With the increase of the surface profile errors, the side effects on the intensity distribution of the beam outputs became more and more obvious, indicated by a spot with non-uniform intensities. For type I and type IV errors, the output light was shown to gradually converge to the center along with the increasing values of the surface profile error. On the contrary, for type II and type III errors, the output light was shown to gradually deviate from the center along with the increasing values of the surface profile error. When the values of the surface profile errors were identical for these four types of situations, type III and IV errors exerted more influence than type I and II errors (see Figure 3c1–c4).

In order to quantitatively compare the influences of four types of surface profile errors on beam homogeneity, a key parameter η was defined as shown in Equation (7).

(7) $$\eta =\frac{I_{\max}-I_{\min}}{I_{\max}+I_{\min}}\times 100\%$$

where *I*_max_ and *I*_min_ represent the maximal and minimal intensities within the output spot, neglecting the effect of the periodic interference pattern.

**Figure 4:**
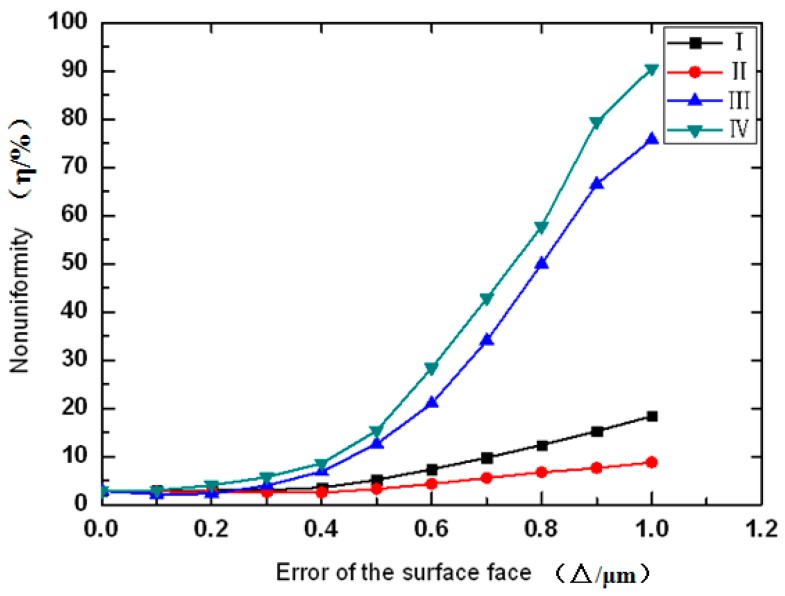
Non-uniformity η as a function of maximal surface profile errors (0–1 μm) of four types of profile mismatches.

The non-uniformities of the surface profile errors within 1 μm were calculated in simulations, as shown in Figure 4. With an increase in the maximal surface profile error △, non-uniformity η was also shown to increase. Again, errors of type III and IV demonstrated more significant effects on beam homogeneity than the other two types of errors. More specifically, a sudden increase in the slope of the non-uniformity metric of 0.4 was observed for type III and IV errors and a further increase of errors led to rapid increases of non-uniformities.

## 4. Experiments and Discussion

**Figure 5:**
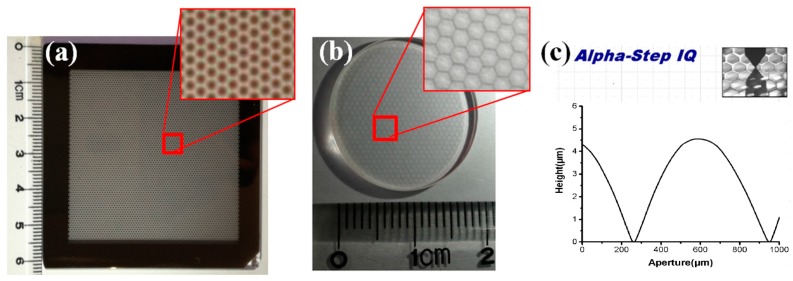
Key fabrication results, including (**a**) gray-scale mask; (**b**) prototype; (**c**) surface profiles.

In order to validate the simulation results, the corresponding experiments were conducted where silica was chosen as the substrate and AZ9260 (AZ Electronic Materials, Somerville, MA, USA) was used as the photoresist, which was spin-coated on the substrate at a speed of 3000 rpm for 20 s. Key parameters of the prebake temperature, the prebake period, and the obtained photoresist thickness were 100 °C, 30 min, and 9 μm, respectively. A gray-scale mask was fabricated to modulate the dose of exposure according to the surface profile of the MLAs (see Figure 5a). After exposure and development, photoresist-based MLAs were obtained. Then the etching of silica was conducted, which transferred the pattern of the MLAs to the silica substrate (see Figure 5b). A step profilometer (ALPHA-Step IQ, KLA-Tencor, Milpitas, CA, USA) was used to measure the surface profiles, validating the spherical structure of the MLAs (see Figure 5c).

**Figure 6:**
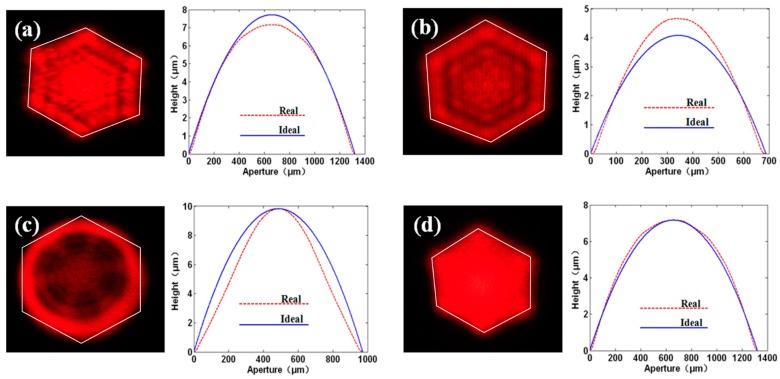
Experimental results of images processed by MLAs with different surface profile errors. The maximal errors were quantified as (**a**) △ = 0.55 μm (type I); (**b**) △ = 0.60 μm (type II); (**c**) △ = 1.78 μm (type III); and (**d**) △ = 0.32 μm (type IV).

A variety of MLAs fabricated with different surface profile errors were used to homogenize the beams, and the corresponding intensity distributions of beams after processing were captured and compared. The corresponding experimental results are shown in Figure 6, where the dashed and solid lines represented the cross-section profiles of fabricated and ideal microlenses, respectively. The maximal surface profile errors in Figure 6a–d were 0.55, 0.60, 1.78 and 0.32 μm, respectively. As to the error types, Figure 6a–d demonstrated four types of errors belonging to type I, II, III and IV, respectively. The theoretical boundary of the expected outer envelope of the irradiated region was drawn with lines in a white color on each image, as shown in Figure 6. 

This deviation of beam homogenization can be explained as follows. The input beam was firstly segmented into several sub-beams by the microlens and then the sub-beams were overlapped with each other through the Fourier lens, generating a uniform intensity distribution. The surface profile errors of the fabricated microlenses led to the irregular distributions of sub-beams, resulting in the redistribution of the divergence angles. Therefore, the output intensity was no longer uniform, and was affected by the fidelities of the surface profiles of microlenses.

If the fabricated microlens showed a type I, surface profile error the curvature of the surface profiles around the light axis at the central region of the microlens was shown to reduce. Therefore, the refractive angle of the light in the corresponding region decreased compared with the light in the same region of the ideal microlens. Since the scale of the light with smaller refractive angles increased, the intensity converged by the Fourier lens was significantly enhanced in the center of the output spot. On the contrary, if the fabricated microlens suffered from a type II surface profile error, the refractive angle of the light in the corresponding region was shown to increase because of the increase of the curvature of the surface profile. Therefore, the intensity was reduced in the center of the output spot because of the decreases in both light intensities and refractive angles.

In addition, when the fabricated microlens demonstrated a type III surface profile error, the curvature of the surface profile firstly increased and then decreased along the direction from the center to the edge of the microlens, compared with the ideal surface profiles. Then the amount of the light with smaller or bigger refractive angles decreased. Therefore, the intensity which should converge at the center and edge of the output spot was translated to regions between the center and the boundary areas. Thus, the intensities at the central and marginal regions were very weak. On the contrary, when the fabricated microlens showed a type IV surface profile error, the corresponding curvature of the surface profile changed in the opposite direction. Therefore, the intensity which should converge in the region between the central and the boundary areas of the output spot was modulated into the regions of the center and the edges. 

Overall, the type III and IV surface profile errors nearly changed the whole surface of the microlens while type I and II surface profile errors only affected one half of the microlens. Therefore, the type III and IV surface profile errors demonstrated more significant influences on beam homogenization. In order to realize beam homogenization, the surface profile errors have to be controlled within a certain range at which the side influences can be tolerated. Meanwhile, the type III and IV surface profile errors should be avoided because of their significant side effects.

## 5. Conclusions

In conclusion, in this study, effects of surface profile mismatches between fabricated and ideal MLAs on beam homogenization were compared and studied. Both numerical simulations and experimental results quantitatively located the side effects of surface profile errors. These results suggest profile deviations located further away from the apex of the microlenses should be avoided in the future fabrication of MLAs since they demonstrated more significant side effects in beam homogenization in comparison to profile errors located on the apex of the microlenses. Hopefully, this study can provide references for the future study of beam homogenization–leveraging microlens arrays.
